# Predictive analysis of the number of human brucellosis cases in Xinjiang, China

**DOI:** 10.1038/s41598-021-91176-5

**Published:** 2021-06-01

**Authors:** Yanling Zheng, Liping Zhang, Chunxia Wang, Kai Wang, Gang Guo, Xueliang Zhang, Jing Wang

**Affiliations:** 1 College of Medical Engineering and Technology, Xinjiang Medical University, Urumqi, 830054 People’s Republic of China; 2 State Key Laboratory of Pathogenesis, Prevention and Treatment of High Incidence Diseases in Central Asia, Clinical Medicine Institute, the First Affiliated Hospital of Xinjiang Medical University, Urumqi, 830054 People’s Republic of China; 3 Department of Respiratory Medicine, First Affiliated Hospital of Xinjiang Medical University, Urumqi, 830054 People’s Republic of China

**Keywords:** Diseases, Mathematics and computing

## Abstract

Brucellosis is one of the major public health problems in China, and human brucellosis represents a serious public health concern in Xinjiang and requires a prediction analysis to help making early planning and putting forward science preventive and control countermeasures. According to the characteristics of the time series of monthly reported cases of human brucellosis in Xinjiang from January 2008 to June 2020, we used seasonal autoregressive integrated moving average (SARIMA) method and nonlinear autoregressive regression neural network (NARNN) method, which are widely prevalent and have high prediction accuracy, to construct prediction models and make prediction analysis. Finally, we established the SARIMA((1,4,5,7),0,0)(0,1,2)^12^ model and the NARNN model with a time lag of 5 and a hidden layer neuron of 10. Both models have high fitting performance. After comparing the accuracies of two established models, we found that the SARIMA((1,4,5,7),0,0)(0,1,2)^12^ model was better than the NARNN model. We used the SARIMA((1,4,5,7),0,0)(0,1,2)^12^ model to predict the number of monthly reported cases of human brucellosis in Xinjiang from July 2020 to December 2021, and the results showed that the fluctuation of the time series from July 2020 to December 2021 was similar to that of the last year and a half while maintaining the current prevention and control ability. The methodology applied here and its prediction values of this study could be useful to give a scientific reference for prevention and control human brucellosis.

Brucellosis is a neglected zoonosis. It causes acute febrile illness and a potentially debilitating chronic infection in humans, and livestock infection has substantial socioeconomic impact^1,2^. *Brucella* spp. can spread through damaged skin, mucous membrane, digestive tract, and respiratory tract^3^. Contact with contaminated animals and consumption of unpasteurized dairy products are the main routes for human infection. Fever, fatigue, hyperhidrosis, muscle and joint pain, spleen, and lymphadenopathy were the main manifestations during the acute period of patient. After a patient is infected, this disease often lasts several weeks or months. If timely treatment can be carried, the patient can be healed, otherwise, become a chronic patient suffering from long-term pain, even lose labor and fertility.

Brucellosis is one of the main diseases endangering public health and safety in China^4–7^. Human brucellosis cases in China, mainly found in pastoral areas, are mostly herdsmen and veterinarians. According to data from the China Public Health Data Center (http://www.phsciencedata.cn/Share/), the first five provinces with the highest incidence of human brucellosis in China are Inner Mongolia, Xinjiang, Ningxia, Shanxi, and Heilongjiang, among which Xinjiang ranks second. A large number of brucellosis patients brought serious harm to the development of social and economic health in Xinjiang. In order to develop measures for the prevention and control of brucellosis in Xinjiang, some activities have been gradually introduced, such as introduction of diagnostic criteria, treatment protocols, control measures, and vaccination of domestic animals. At present, the annual number of human brucellosis cases was still very large. Therefore, the prevention and control of brucellosis in Xinjiang need continuous efforts, and the task of prevention and control is still arduous.

Scientific analysis and prediction of the number of human brucellosis cases can provide recommendations for evaluating and formulating health financing policies, health reform, and development strategies. Prediction techniques include quantitative and qualitative forecasting. Quantitative forecasting requires hard data and number crunching, while qualitative forecasting relies more on estimates and instincts of experts. Compared to qualitative forecasting, quantitative forecasting is more widely used and requires statistical methods and mathematical methods including differential equation method^8–11^, time series prediction method^12–14^, regression method^15,16^, neural network method^17–22^, and grey prediction method^23,24^, and so on. Among which the NARNN model of the neural network methods has high prediction performance and can capture the nonlinear information of the data well^25–27^, and the SARIMA model of time series methods can capture the periodicity, trend, and randomness of the data well^28^. Over the past several years, both NARNN model and SARIMA model were widely used in the prediction of various diseases incidence. For example, Saba et al^29^ forecasted the prevalence of COVID-19 outbreak in Egypt using nonlinear autoregressive artificial neural networks(NARNN). Zhou et al^30^ used the SARIMA, the NARNN, and the hybrid SARIMA-NARNN model to fit and forecast the monthly and daily number of new admission inpatients. Ebhuoma et al^31^ predicted monthly incidence of malaria using SARIMA model. Xu et al.^32^ forecasted the incidence of Mumps in Zibo City based on a SARIMA Model; etc^33–38^.

In this article, we aimed to make a prediction analysis of the number of human brucellosis cases based on an academic point of view so as to help to make early planning for the future situation and put forward more scientific preventive and control countermeasures. The paper is structured in the following way: first, we established an optimal SARIMA model to predict and analyze the number of human brucellosis cases in Xinjiang. Second, we established an optimal NARNN model to predict and analyze the number of human brucellosis cases in Xinjiang. Third, we compared the prediction performance of the two models. Finally, the better model was chosen to do prediction analysis from July 2020 to December 2021.

## Materials and methods

### Data sources

We collected the annual incidence of human brucellosis of 31 provinces in China from 2014 to 2017 and the number of monthly reported cases of human brucellosis in Xinjiang from January 2008 to June 2020. Among which the data from January 2008 to December 2017 were from the website of the National Public Health data Center (http://www.phsciencedata.cn/Share/), and the data from January 2018 to June 2020 were from the website of the Xinjiang Health and Health Commission. The number of reported annual cases of human brucellosis in Xinjiang from 2018 to 2019 was shown in Table 1.

**Table 1:** The number of reported annual cases of human brucellosis in Xinjiang from 2008 to 2019.

Year	Reported cases	Year	Reported cases
2008	425	2014	7708
2009	498	2015	8467
2010	848	2016	8042
2011	1411	2017	6485
2012	2335	2018	4429
2013	4095	2019	4349

### SARIMA model^39^

In the fields of prediction, we often encounter non-stationary time series with seasonality, trend and randomness. The SARIMA model is very suitable for predicting and analyzing this kind of time series^28^. The SARIMA model is derived from the autoregressive integrated moving average model (ARIMA) created by Box and Jenkins. It is a short-term prediction method with high accuracy^39^. It can realize the structural characteristics of time series more fundamentally and achieve the optimal prediction in the sense of variance^39^. When the time series studied are seasonal, the ARIMA model often requires some seasonal processing, which transforms the ARIMA model into a SARIMA(p,d,q)(P,D,Q)_s_ model, and it has the following forms:

$$\begin{aligned} & (1 - \varphi_{1} L - \cdots - \varphi_{p} L^{p} )(1 - \alpha_{1} L^{s} - \cdots - \alpha_{P} L^{Ps} )(\Delta^{d} \Delta_{s}^{D} y_{t} ) \\ & \quad = (1 + \theta_{1} L + \cdots + \theta_{q} L^{q} )(1 + \beta_{1} L^{s} + \cdots + \beta_{Q} L^{Qs} )\varepsilon_{t} , \\ \end{aligned}$$

$$\begin{aligned} & \Delta_{s}^{1} y_{t} = (1 - L^{s} )y_{t} = y_{t} - y_{t - s} , \\ & (1 - L)y_{t} = y_{t} - y_{t - 1} , \\ \end{aligned}$$

where,$\alpha_{{1}} ,\alpha_{{2}} , \ldots ,\alpha_{P}$, $\beta_{{1}} ,\beta_{{2}} , \ldots ,\beta_{Q}$,$\varphi_{{1}} ,\varphi_{{2}} , \ldots ,\varphi_{p}$,$\theta_{{1}} ,\theta_{{2}} , \ldots ,\,\,{\text{and}}\,\,\theta_{q}$ are parameters. $\Delta^{{\text{d}}}$ means doing d times ordinary differencing, and $\Delta_{s}^{D}$ means doing D times season differencing. Seasonal cycle s of monthly time series is 12.

In particular, if some parameters such as $\alpha_{{2}}$, $\beta_{{2}}$, $\varphi_{{2}}$, $\theta_{{2}}$ in the model are 0, then the SARIMA(p,d,q)(P,D,Q)s model becomes sparse. This sparse SARIMA(p,d,q)(P,D,Q)s model can be characterized as:

$${\text{SARIMA}}((1,3, \ldots ,p),{\text{d}}, \, (1,3, \ldots ,q))((1,3, \ldots ,P),{\text{D}}, \, (1,3, \ldots ,Q))_{{\text{s}}}$$

In this study, we collected the data of monthly reported cases of human brucellosis in Xinjiang from January 2008 to June 2020. The data had obvious seasonality, trend and randomness. According to the characteristics of the data, we chose the SARIMA method to do prediction analysis.

The construction process of SARIMA model includes: 1. To Make the research sequence stable by ordinary differencing or seasonal differencing. The d is the degree of ordinary differencing, and the D is the degree of seasonal differencing. 2. To determine possible P, Q, p, and q by analyzing the autocorrelation function (ACF) graph and partial correlation function (PACF) graph of stationary sequence. This step requires researcher’s professional knowledge and experience. 3. To test the parameters of the possible models (p-value less than 0.05 means statistical significance), and then to compare their Akaike Information Criterion (AIC), Schwarz Criterion (SC), and R^2^ of models passed parameter tests. The larger the R^2^ is, and the smaller the AIC and SC values are, the higher the goodness of fit of the model is. 4. To do the residual error test of the established model with the largest R^2^, smallest AIC and SC. If there is almost no autocorrelation and partial correlation among the residuals of the model, then the residual error will be white noise, indicating that the established model has extracted the original information fully and can be successfully used for prediction analysis.

### Nonlinear Autoregressive Neural Network (NARNN)^40–42^

In recent years, the NARNN has achieved good results in time series analysis because of its good nonlinear characteristics, parallel distributed storage structure and high fault tolerance. As a kind of dynamic neural network, the NARNN has good dynamic and anti-interference ability, and can be used to approximate any nonlinear dynamic system. The NARNN model is a dynamic neural network model based on time series, it takes into account the nonlinear ability of neural network and the advantages of autoregressive (AR) model in processing time series. The output of each time is based on the dynamic results of the system before the current time, and it has the function of feedback and memory. The NARNN function model can be expressed as:

$$y(t) = f[y(t - 1),y(t - 2), \ldots ,y(t - n)],$$

where, *y* represents input parameter, t denotes time period, n is the delay order of the NARNN model, and the $f[ \cdot ]$ is the function of the NARNN model. The previously obtained health state sequence F(t) is used as the input of the NARNN to establish a prediction model for the time series.

In this work, to develop the optimal NARNN model, the monthly reported number of human brucellosis cases from January 2008 to June 2020 was randomly broken into three subseries: the training, validation, and testing subseries comprising 70%, 15%, and 15% of all the data, respectively. A series of experiments with different hidden neurons and d delays were then conducted to find the optimal method in an open feedback mode based on the autocorrelogram, response graph of the outputs and targets, mean square error^43^. Lastly, a prediction into the testing series can be conducted in a closed-loop mode.

### The measurement of model comparison

Root mean square error (RMSE), mean absolute error (MAE), and mean absolute percentage error (MAPE) are often used to measure the model fitting performance^43^. In this study, the RMSE, MAE, and MAPE are used to compare the fitting ability of the established SARIMA model and the NARNN model. The smaller these values are, the better the fitting and prediction performance of model is. The measurements are expressed as^43,44^:

$$\begin{aligned} RMSE & = \sqrt {\frac{{\mathop \sum \nolimits_{t = 1}^{n} \left( {y_{t - } \widehat{{y_{t} }}} \right)^{2} }}{n}} , \\ MAE & = \frac{{\mathop \sum \nolimits_{t = 1}^{n} \left| {y_{t - } \widehat{{y_{t} }}} \right|}}{n}, \\ MAPE & = \frac{{\mathop \sum \nolimits_{t = 1}^{n} \left| {\frac{{y_{t - } \widehat{{y_{t} }}}}{{y_{t} }}} \right| \times 100}}{n}, \\ \end{aligned}$$

where, $\widehat{{y_{t} }}$ is the predicted value of *y*_*t*_, *y*_*t*_ is the actual values, and *n* is the number of observations.

### Statistical software

**Figure 1:**
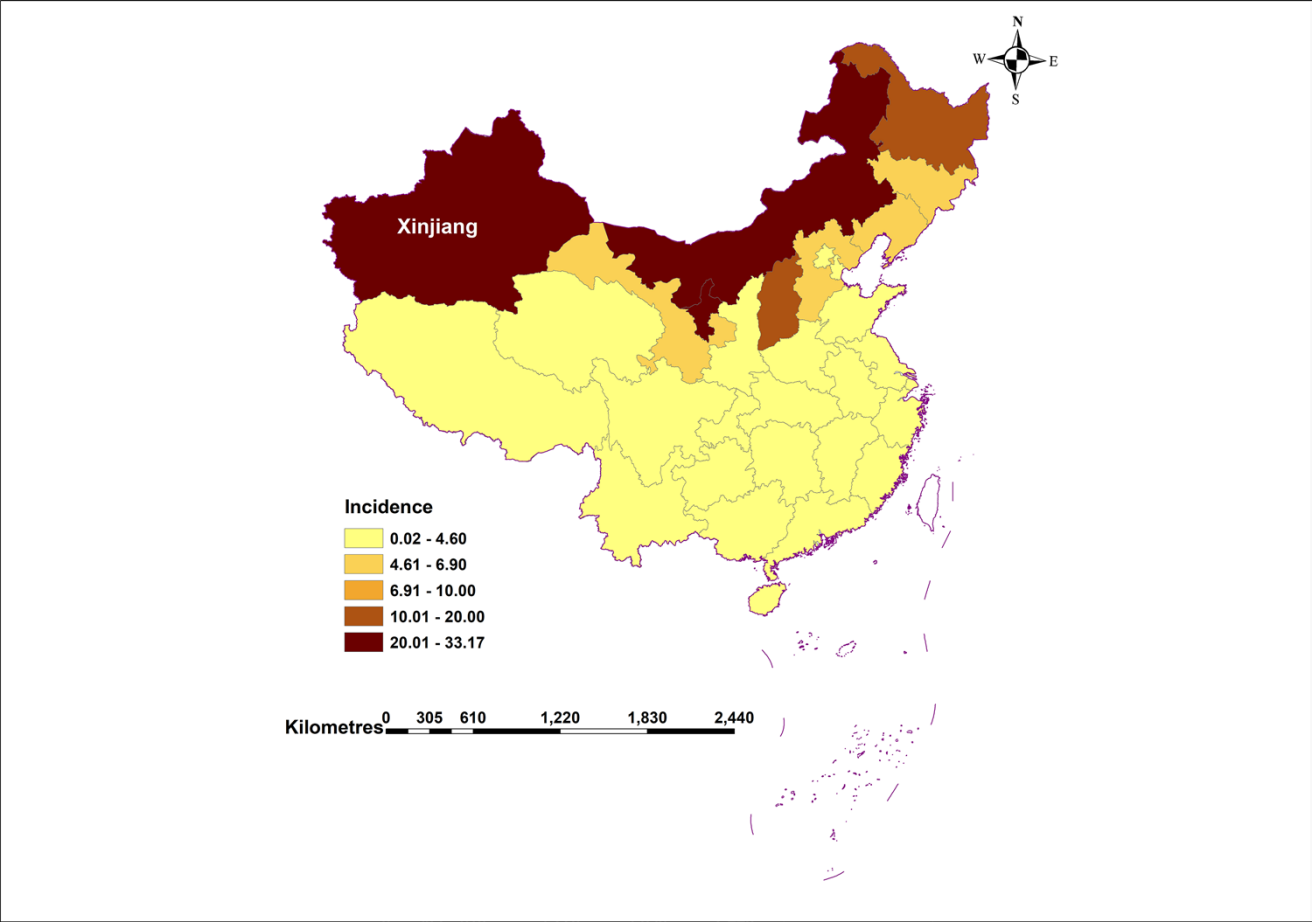
The average annual incidence (per 100,000 populations) of human brucellosis in China from 2014 to 2017. (This figure is plotted by ArcMap10.4.1).

We conducted SARIMA models in R [v3.6.2] using the forecast package, and Eviews7.0 software. We conducted NARNN models by matlab2012b. Except for Fig. 1 plotted by ArcMap10.4.1, all other Figures are plotted by matlab2012b.

## Results

**Figure 2:**
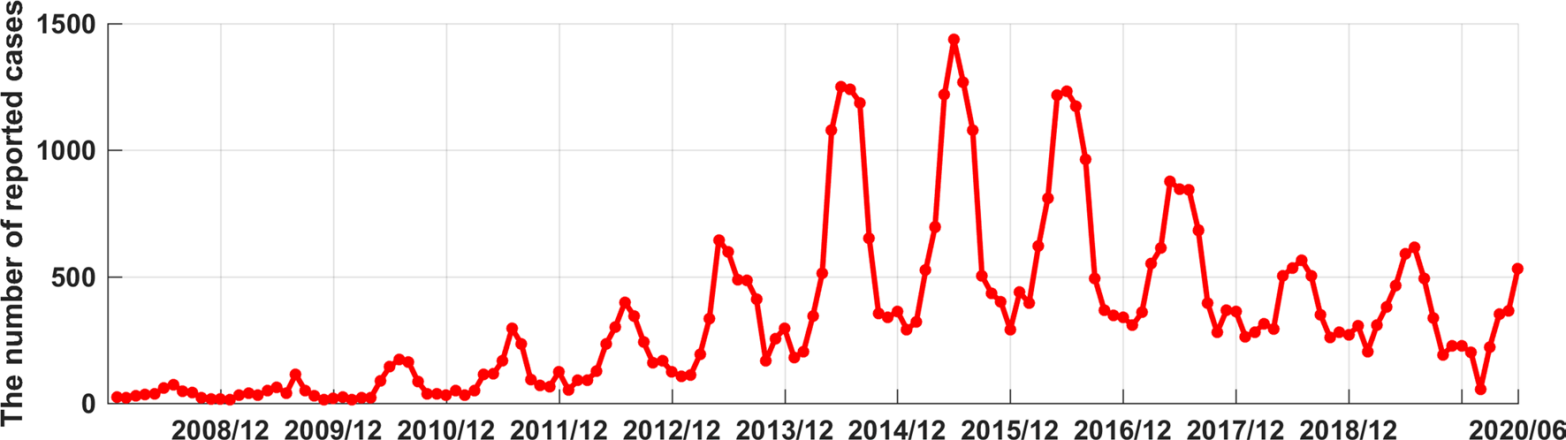
The time series of monthly reported cases of human brucellosis in Xinjiang from January 2008 to June 2020.

Drawing the distribution graph (Fig. 1) of the average annual incidence of human brucellosis in China from 2014 to 2017. From which we can see that the incidence of human brucellosis in Xinjiang was higher than that of most provinces. From January 2008 to June 2020, 51,182 cases of human brucellosis were reported in Xinjiang, and the number of reported cases in this period was shown in Fig. 2. From Fig. 2, it could be seen that the time series of human brucellosis cases was obviously seasonal. From May to August of each year, it was the high incidence period of this disease. From 2008 to 2015, the number of human brucellosis cases showed an upward trend. After that, under the vigorous prevention and control of the government and Centers for Disease Control and Prevention in Xinjiang, the number of the brucellosis patients decreased year by year. In February last year, in the case of strict prevention of COVID-19, the incidence of this disease had also been greatly controlled.

### The prediction analysis of SARIMA model

**Figure 3:**
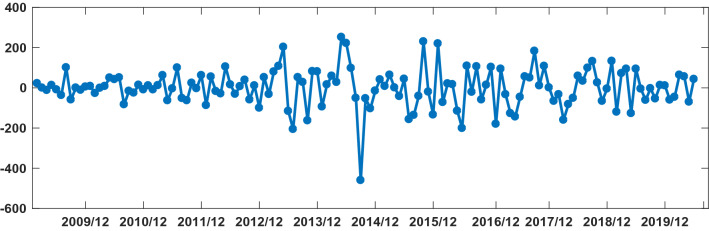
Plot of time series after differencing.

**Figure 4:**
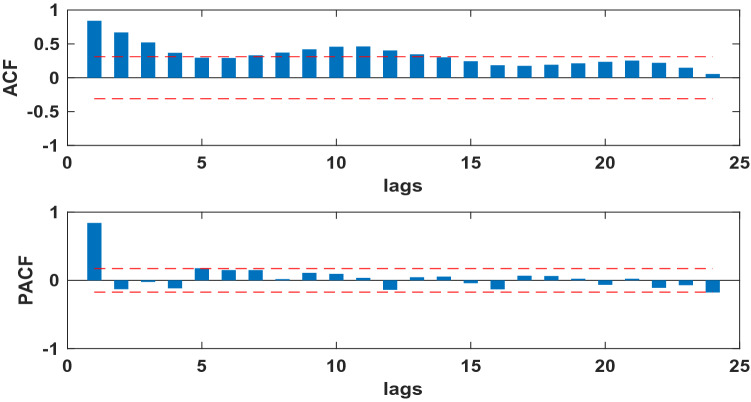
The ACF and PACF of time series after differencing of human brucellosis cases in Xinjiang.

**Figure 5:**
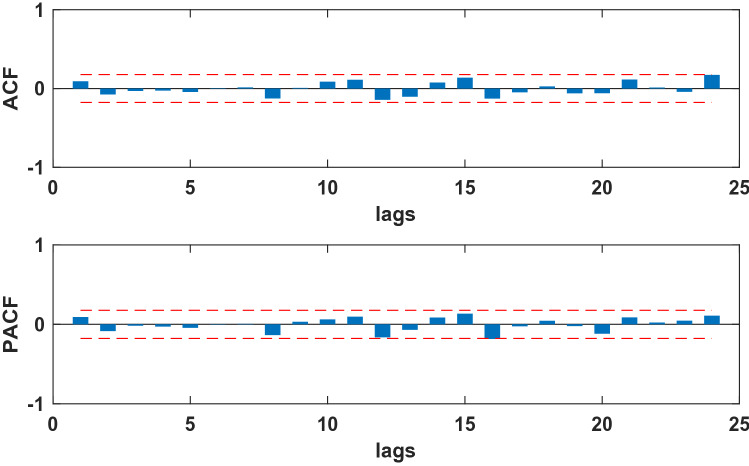
The ACF and PACF of residual sequence of the SARIMA((1,4,5,7),0,0)(0,1,2)_12_ model.

The SARIMA model is based on stationary data. Firstly, we used Augmented Dickey Fuller's test (ADF) to test the stationarity of the data. The p**-value** of the test was 0.56, which indicated that the data was not stationary. Because the data had obvious seasonality (s = 12), we did seasonal differencing to make the data stationary. After the seasonal differencing, the p-value of the ADF test is less than 0.05, and transformed time series appeared to be stationary (see Fig. 3), indicating that the data has been stationary (d = 0, D = 1). Draw the ACF and PACF graphs (see Fig. 4) of the stationary data to help us determining these values of possible p, q, P, and Q. Based on the analysis of Fig. 4, we found that the autocorrelation coefficients were trailing, therefore, we gave q = 0, Q = 1 or 2. For the value of p, we tried taking 1, 2, 4, 5, 6, and 7, respectively. For the value of P, we took 1. Then, the parameters of SARIMA(p,0,0)(P, 1, Q)_12_ models with different combination of p, P, and Q were tested, and the AIC and SC values of these models were calculated. Finally, only six models (SARIMA(1,0,0)(0,1,0)_12_, SARIMA((1,4,7),0,0)(0,1,0)_12_, SARIMA((1,5,7),0,0)(0,1,0)_12_,SARIMA((1,4,5),0,0)(0,1,2)_12_,SARIMA((1,4,7),0,0)(0,1,2)_12_,and SARIMA((1,4,5,7),0,0)(0,1,2)_12_) passed all parameter tests, as shown in Table 2. Of these six models, model 6 had the smallest AIC and the largest R^2^, so it was the model with the best fitting ability. The expression of model 6 was SARIMA((1,4,5,7),0,0)(0,1,2)_12_. In order to examine the residuals of the SARIMA((1,4,5,7),0,0)(0,1,2)_12_ model, we drew the ACF and PACF plots of the model residuals (see Fig. 5). It could be seen from Fig. 5 that the autocorrelation and partial correlation coefficients of the residuals **were** basically within twice the standard deviation, indicating that the residuals **were** basically white noise, and the SARIMA((1,4,5,7),0,0)(0,1,2)_12_ model extract**ed** the information of the original data well and had good performance. Therefore, the SARIMA((1,4,5,7),0,0)(0,1,2)_12_ model c**ould** be used to predict the number of reported cases of human brucellosis in Xinjiang.

**Table 2:** Results of parameter tests and the AIC, SC, and R^2^ values of the six models of passed parameter tests.

	Model 1	Model 2	Model 3	Model 4	Model 5	Model 6
AR(1)	0.85*	0.89*	0.84*	0.95*	0.9*	0.93*
AR(4)	–	− 0.18*	–	− 0.4*	− 0.18*	− 0.33*
AR(5)	–	–	− 0.13*	0.33*	–	0.2*
AR(7)	–	0.17*	0.18*	–	0.19*	0.12*
SMA(2)	–	–	–	− 0.89*	− 0.88*	− 0.89*
AIC	11.85	11.83	11.87	11.58	11.59	11.57
SC	11.88	11.9	11.93	11.67	11.67	11.68
R^2^	0.71	0.74	0.73	0.794	0.796	0.802

*Means significant at 0.05 level.

### The prediction analysis of NARNN model

**Figure 6:**
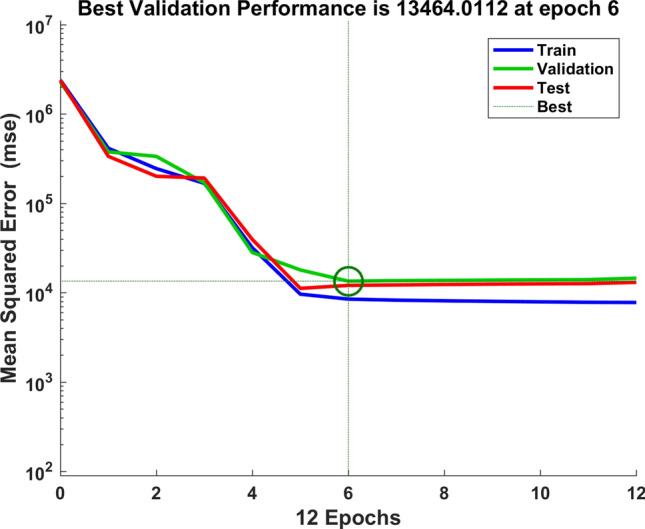
The graph of looking for the best validation performance.

**Figure 7:**
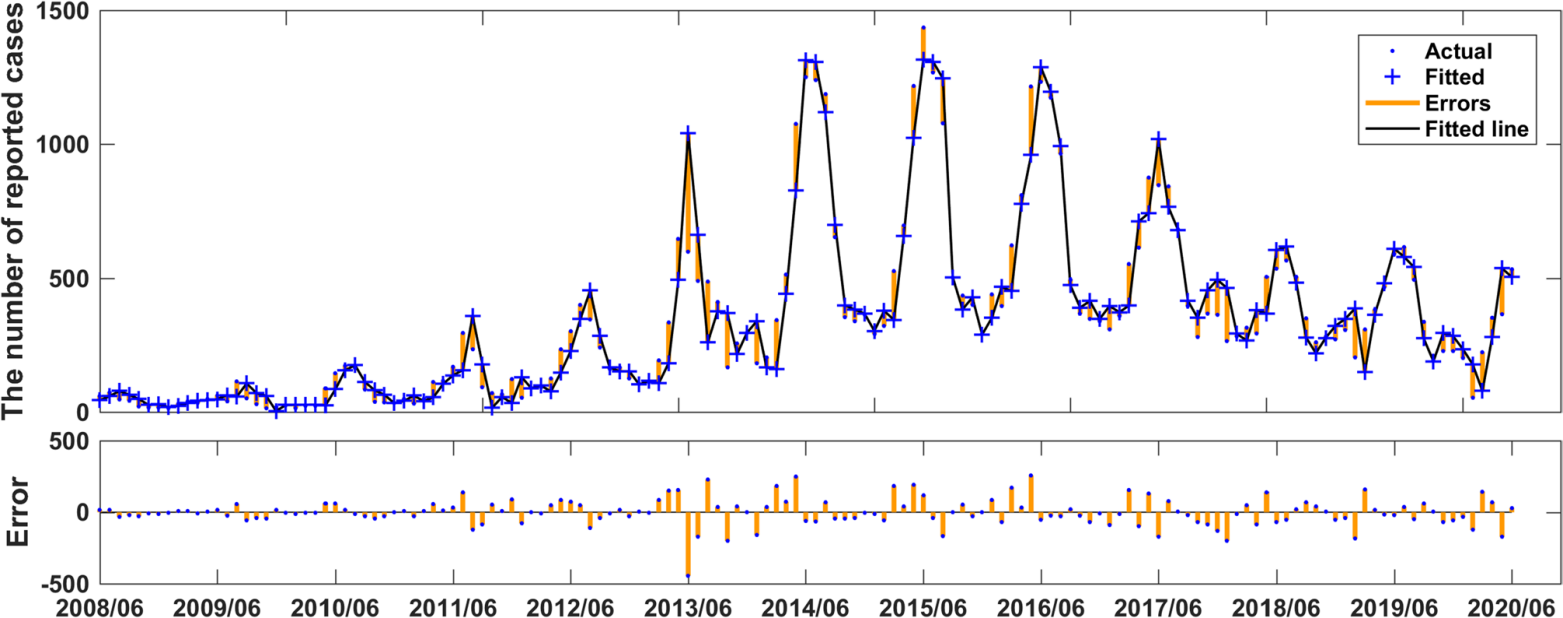
Actual series, fitted series, and residual series of the reported cases sequence of human brucellosis.

**Figure 8:**
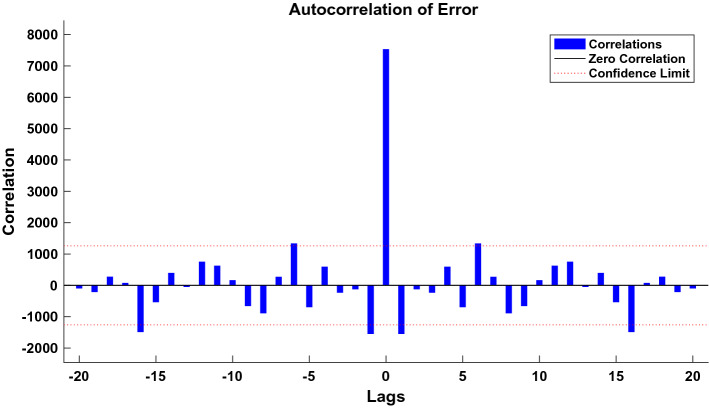
Autocorrelation graph of the residuals of the NARNN model.

We used the data of time series of human brucellosis cases from January 2008 to June 2020 in Xinjiang to train NARNN, 70% of the raw data was used as training data, 15% as validation data, and the remaining 15% as test data. By repeatedly adjusting the number of neurons in the hidden layer and the number of time lags, and finally, we found that the NARNN structure with 10 hidden layer neurons and 5 time lag was the best, and its error requirement was satisfied. The best validation performance was 13,464.0112 at epoch 6 (see Fig. 6). By using the established NARNN model to fit the original the number of monthly reported cases of human brucellosis, we got the graph of fitting and error results (see Fig. 7). The autocorrelation diagram of the errors was shown in Fig. 8. From Fig. 8, we could see that the error was only the largest correlation with itself, and the correlation coefficient at other lags was almost in the confidence interval, indicating that the established NARNN model had good fitting performance.

### Model comparison

**Figure 9:**
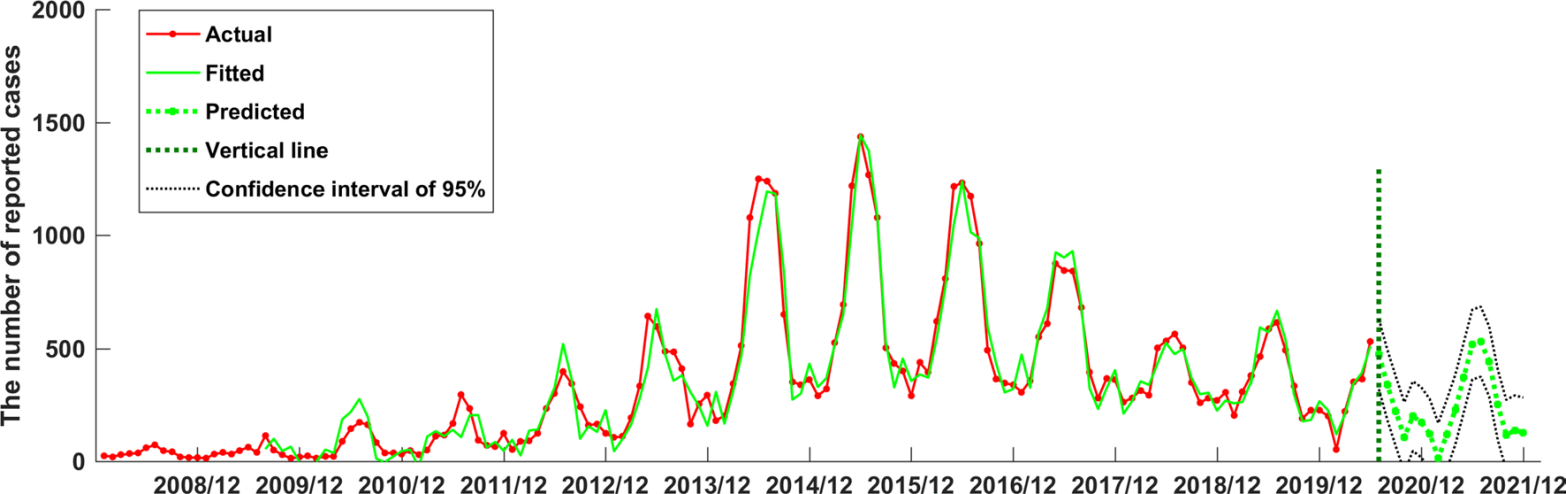
The SARIMA((1,4,5,7),0,0)(0,1,2)_12_ model fitted and forecasted time series of reported cases of human brucellosis in Xinjiang.

Both the SARIMA((1,4,5,7),0,0)(0,1,2)_12_ model and the established NARNN model in this study had good fitting performance and could be used to predict and analyze the number of reported cases of human brucellosis in the future. However, in order to obtain more accurate prediction values, we aimed to compare the two models and selected the better one to make prediction analysis. Therefore, we calculated the RMSE, MAE, and MAPE of the two models when they fitted the original human brucellosis sequence, respectively (see Table 3). From Table 3, we could see that the RMSE, MAE, and MAPE of the SARIMA((1,4,5,7),0,0)(0,1,2)_12_ model were smaller than that of the NARNN model, indicating that the SARIMA((1,4,5,7),0,0)(0,1,2)_12_ model was better than the established NARNN model. Therefore, the SARIMA((1,4,5,7),0,0)(0,1,2)_12_ model was more suitable to predict the future number of human brucellosis cases in Xinjiang. We used the SARIMA((1,4,5,7),0,0)(0,1,2)_12_ model to predict the number of reported cases of human brucellosis in Xinjiang from July 2020 to December 2021, as shown in Table 4. Furthermore, in order to see the performance of fitting and prediction more intuitively, we plotted the Fig. 9.

**Table 3:** Fitting accuracy values of the SARIMA((1,4,5,7),0,0)(0,1,2)_12_ model and the NARNN model.

	RMSE	MAE	MAPE
SARIMA((1,4,5,7),0,0)(0,1,2)^12^	75.87	56.62	32.4
NARNN	92.46	61.47	33.6

**Table 4:** The number of predicted human brucellosis cases in Xinjiang by the SARIMA((1,4,5,7),0,0)(0,1,2)_12_ model.

Date	Predicted	Date	Predicted	Date	Predicted
2020/07	476	2021/01	121	2021/07	531
2020/08	340	2021/02	15	2021/08	443
2020/09	221	2021/03	120	2021/09	252
2020/10	108	2021/04	230	2021/10	118
2020/11	201	2021/05	374	2021/11	137
2020/12	174	2021/06	519	2021/12	128

## Discussion

Brucellosis remains a serious public health issue, and more efforts are needed to reach the goal of controlling human and animal brucellosis in China. Xinjiang has a high incidence of human brucellosis, and the brucellosis patients are mainly pastoralists and veterinarians. The total population of Xinjiang is no more than thirty million, furthermore, the number of herdsmen is not very large, but the number of monthly reported cases of human brucellosis is relatively large. It shows that the incidence of human brucellosis is very high in herdsmen and veterinary groups. From 2008 to 2016, the incidence of human brucellosis in Xinjiang increased year by year, which caused great harm to Xinjiang's economic construction and the development of animal husbandry. In recent several years, the relevant departments of disease prevention and control in Xinjiang have stepped up their prevention and control efforts; therefore, the number of human brucellosis cases has been decreasing year by year.

It is well accepted that the accurate identification of seasonality plays a good role in timely responses and reasonably allocated resource for infectious disease^45^. In our study, human brucellosis can occur during all seasons, but a clear seasonality with a periodicity of 12 months was noted from January 2008 to December 2019, and there was a peak in May, June, July, and August of every year, similar seasonal characteristics are also presented in other researches^45,46^. In Xinjiang, a variety of complicated factors may be responsible for human brucellosis peak such as temperature, humidity, wind speed, air pollution, and the climatic characteristics of summer with high temperature affecting people's behavior and diet.

In order to further eliminate brucellosis, the prevention and control technology of brucellosis needs to be gradually improved; and helps from all sides are needed. Forecasting epidemic trend of infectious diseases is an important link of disease prevention and control**.** In this study, we have aimed to do some prediction analysis providing some help for the prevention and control of human brucellosis in Xinjiang. After careful analysis, we established the optimal SARIMA((1,4,5,7),0,0,)(0,1,2)_12_ model. In the construction of the NARNN model, based on the characteristics of the data and repeated debugging, we finally identified the NARNN with a time lag of 5 and a hidden layer neuron of 10. Using RMSE, MAE and MAPE to compare the fitting abilities of the SARIMA((1,4,5,7),0,0,)(0,1,2)_12_ model with that of the established NARNN model, we got the conclusion. The conclusion showed that the SARIMA((1,4,5,7),0,0,)(0,1,2)_12_ model had smaller values of RMSE, MAE, and MAPE, indicating that the fitting ability of the SARIMA((1,4,5,7),0,0,)(0,1,2)_12_ model was better than that of the established NARNN model. As a result, the SARIMA((1,4,5,7),0,0,)(0,1,2)_12_ model was more suitable for future prediction of the number of human brucellosis cases in Xinjiang. We used the SARIMA((1,4,5,7),0,0,)(0,1,2)_12_ model to predict the number of reported cases of human brucellosis in Xinjiang from July 2020 to December 2021. And the results showed that if existing prevention and control efforts are maintained, the changes of the monthly incidence of human brucellosis will be similar to that of July 2019 to December 2020.

Several studies found that the NARNN method has good performance when it is used to do prediction analysis^47^. However, most studies found that SARIMA method has a good ability of fitting and forecasting analysis^31–34^. Based on the characteristics of our data, we also found that the SARIMA method outperforms the NARNN method. From Fig. 9, we can see that the established SARIMA((1,4,5,7),0,0,)(0,1,2)_12_ model can fit history data successfully, and give credible prediction numbers of human brucellosis cases in Xinjiang.

However, in this study, there are also two main limitations. One of the limitations is the access to more granular data such as environmental data, meteorological data, local regulations, and so on. These might allow for a better explanation of changes in the number of human brucellosis cases in Xinjiang. Further improvements of the proposed model may be achieved by incorporating additional information. Another limitation is that the accuracy of long-term prediction of the SARIMA((1,4,5,7),0,0,)(0,1,2)_12_ model will be reduced. Therefore, if long-term prediction with high accuracy is needed, then new data and updating the model further will be required.

## Conclusions

This study is the first prediction analysis of the number of human brucellosis cases in Xinjiang for the period 2008 to 2021. Our study found that both SARIMA method and NARNN method are effective methods for predicting the number of human brucellosis cases in Xinjiang, and the accuracy of the SARIMA method outperforms that of the NARNN method. Using the established SARIMA((1,4,5,7),0,0,)(0,1,2)_12_ model, we predicted the number of human brucellosis cases in Xinjiang from July 2020 to December 2021.Our study method and prediction results can contribute some ideas for the policy maker to make decision in the future.
